# Optimization of Hybrid Composite–Metal Joints: Single Pin

**DOI:** 10.3390/ma18071664

**Published:** 2025-04-04

**Authors:** Ruopu Bian, Bin Wang, Hongying Yang, Jiazhi Ren, Lujun Cui, Oluwamayokun B. Adetoro

**Affiliations:** 1 College of Intelligent Textile and Fabric Electronics, Zhongyuan University of Technology, Zhengzhou 450007, China; 2 Department of Mechanical and Aerospace Engineering, Brunel University London, London UB8 3PH, UK

**Keywords:** optimization, metal to composite joints, finite element analysis, pin joint, stress

## Abstract

Deepening the understanding of composite and metal joint methodologies applied in the aerospace industry is crucial for minimizing operational expenditures. Current investigations are focusing on innovative joining techniques that incorporate additive manufactured rivet pins. This research aims to analyze the mechanical strength of these joints for the effective optimization of pin profiles. Through extensive study of the impact of pin geometry on joint performance, we derived the optimal pin design, considering various initial parameters with the objective of minimizing stress concentration in the pin structure. The joint configurations of metal to composite interfaces were systematically examined using finite element analysis and lap shear testing, which included a singular pin and an adhesive-bonding layer. Numerical simulations reveal that the maximum shear stress in the pin is located at the junction between the base of the pin and the metal plate. By optimizing the shape and dimensions of the pin, both the shear and axial stresses can be significantly mitigated. Following the numerical optimization process, a series of enhanced pins have been produced via additive manufacturing techniques to facilitate mechanical testing. The experimental data obtained align closely with the simulation results, thereby reinforcing the validity of the optimization. The optimal configuration for a single pin, involving a 60° angle and a total height of 3.43 mm, achieves the minimum shear stress. Based on these findings, further investigations are underway to explore optimized designs utilizing multiple pins. This paper presents the results of the single pin study, whereas the findings pertaining to the ongoing investigation on the multi-pin configuration will be disseminated in subsequent publications.

## 1. Introduction

In modern aircraft generations, manufacturers have shifted from using metals (e.g., aluminum alloys and titanium) as the main structural material to composites. For example, the Airbus A350 and Boeing 787 utilize fiber-reinforced composites for more than 50% of their structural weight [1,2,3,4,5]. These composite materials present a multitude of advantages over traditional aluminum alloys, including a significantly high specific strength to weight ratio and diminished sensitivity to fatigue failure. The aviation industry increasingly incorporates both metallic materials, such as aluminum alloys and titanium, as well as composite materials, particularly carbon-fiber-reinforced polymers (CFRPs), in structural joints to fulfill the stringent industry requirements.

In the field of metal to composite joining techniques, mechanical fasteners (e.g., pins) and adhesive bonding are the predominant methods currently utilized. Each of these joining techniques exhibits its own set of advantages and disadvantages [6,7]. One of the primary benefits of adhesive bonding is its ability to unify two dissimilar material plates without inducing physical damage, a significant contrast to riveting, which often compromises the internal integrity of the materials involved. Nevertheless, adhesive bonding is not without its limitations, which include concerns regarding joint durability (material degradation), the necessity for meticulous surface treatments, and constraints imposed by environmental conditions such as humidity and temperature. For instance, during high-altitude flights, aircraft are exposed to low temperatures and elevated humidity levels—conditions that can rapidly diminish the effectiveness of adhesive bonds.

Consequently, the pin-joint process remains a prevalent practice in the maintenance of composite components, serving as a rapid and temporary solution for structural integrity issues. However, when composite plies are riveted, more than 60% of the strength is lost due to the presence of holes, which damages the integrity of the material and causes potential failure without early detection [8,9,10,11]. As the utilization of composite materials in the aerospace sector continues to rise, a comprehensive understanding of the methodologies for joining composites and metals is paramount for improving safety and minimizing costs [12,13,14,15,16].

**Figure 1:**
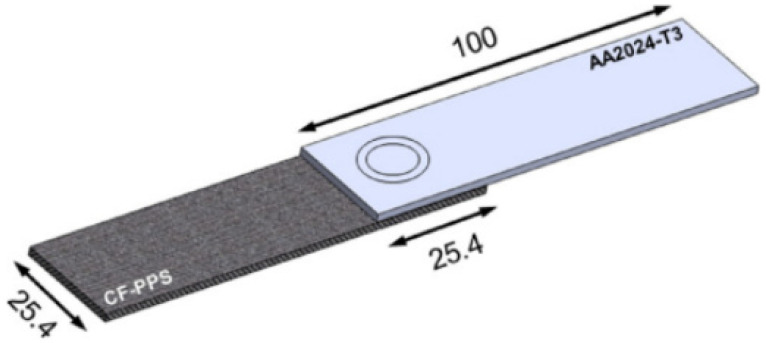
Configuration and dimensions of the friction spot joints (in mm) [17].

**Figure 2:**
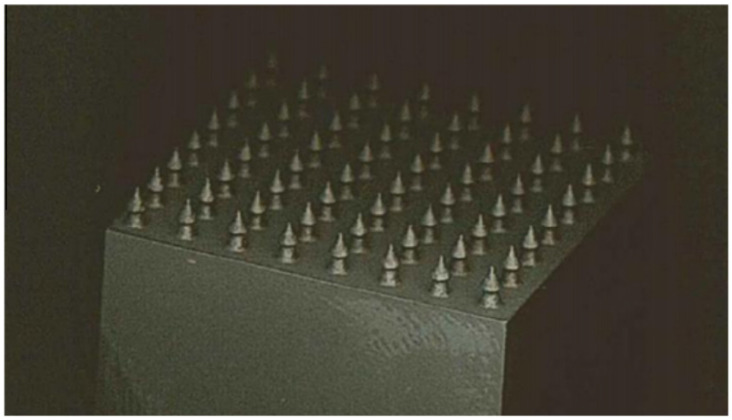
An array of additively manufactured titanium HYPER pins [20].

To tackle this challenge, several joining techniques have been investigated, including friction spot joining (illustrated in Figure 1) [17]. This technique is notable for its ability to achieve high strength while remaining lightweight and cost effective. Additionally, one of the key advantages of selective laser melting is its capability to bypass the necessity for surface preparation [18]. Other methods, such as inducting spot welding and hybrid penetrative reinforcement (Figure 2), showcase rapid heating capabilities and enhanced strength [19,20]. Although various studies have examined configurations involving multiple rows of pins and composite connectors [20], significant gaps remain in the research, particularly concerning the impact of pin geometry and the spatial distribution of multiple pins.

**Figure 3:**
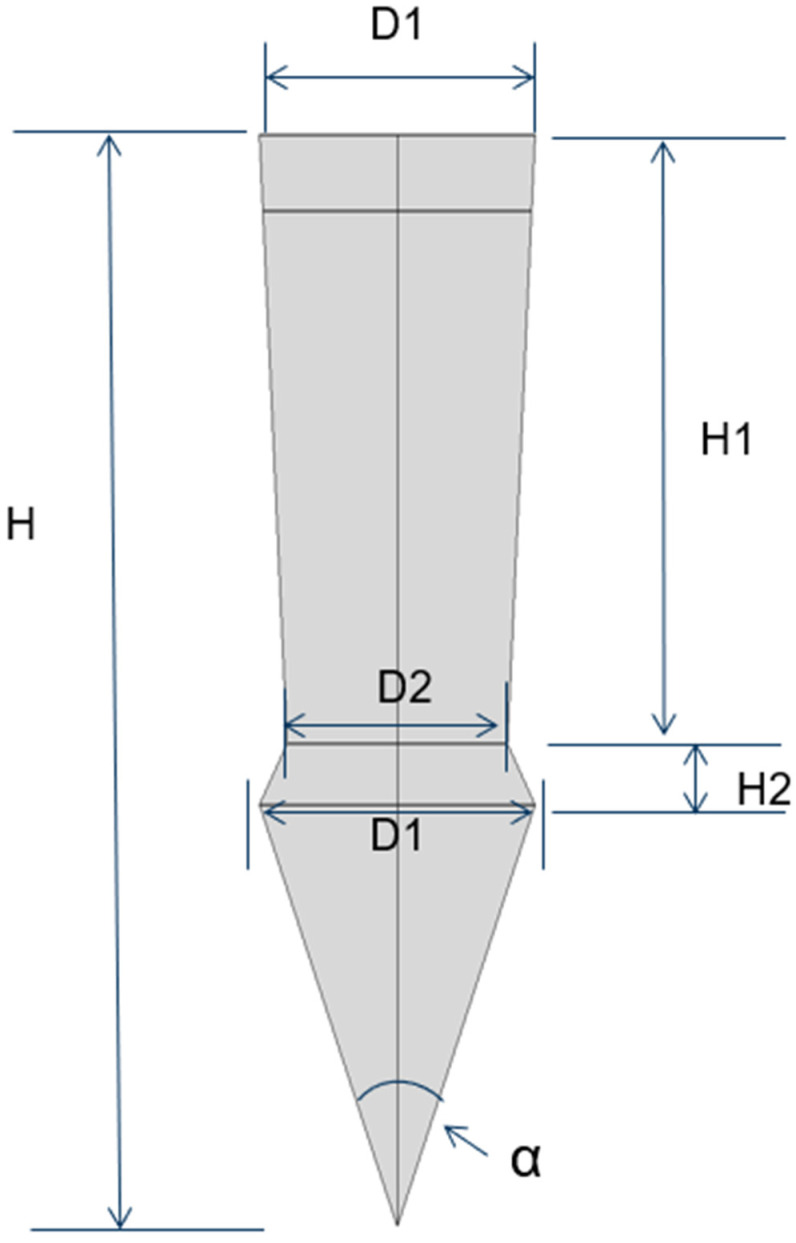
Parameters of the pin.

**Figure 4:**
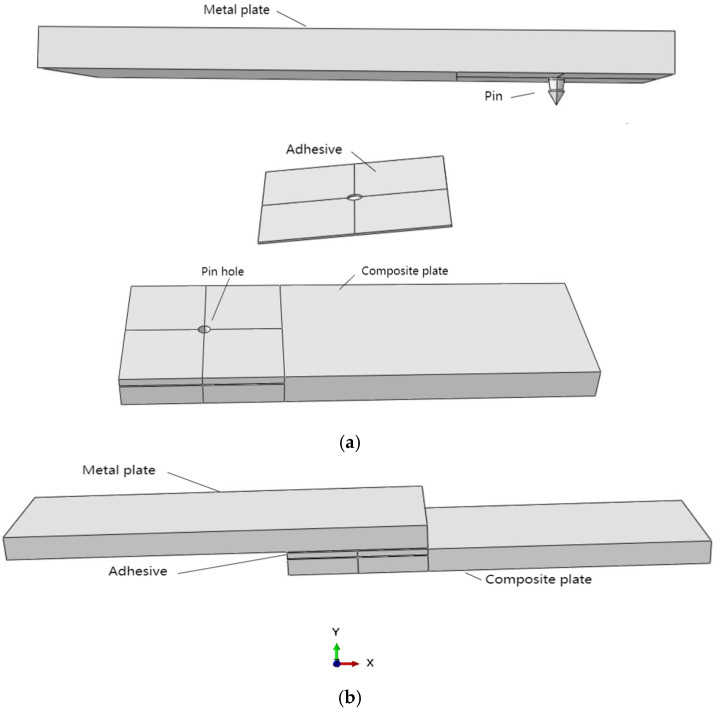
(**a**) Three parts of the model (metal plate, adhesive layer, and composite plate); (**b**) whole model.

This research aims to address these gaps by optimizing both the geometric dimensions and profiles of the pins, as detailed in Figure 3. Furthermore, we will explore the integration of an adhesive layer between the metal plate and the composite plate, as illustrated in Figure 4a. To augment the strength between the metal plate and the pin, we propose the use of additive manufacturing techniques to 3D-print the pins directly onto the metal substrate, thereby enhancing the overall structural integrity of the assembly.

## 2. Methods of Study

This study employed an integrated methodology that combines experimental testing with numerical simulations through finite element modeling The experimental component encompassed the 3D-printing of pins and conducting lap shear tests.

### 2.1. Numerical Analysis

Finite element analysis (FEA) was applied to investigate a metal-to-composite joint featuring a single pin bonded with adhesive. The finite element model consisted of three distinct components: the top metal plate, which houses the pin on its lower surface; an intermediate adhesive layer; and a bottom composite plate, as illustrated in Figure 4b. The modeling was executed using Abaqus (version 6.14, Dassault SIMULIA Company, Providence, RI, USA). The material properties, along with the initial dimensions of the pin, are detailed in Table 1 and Table 2.

**Figure 5:**
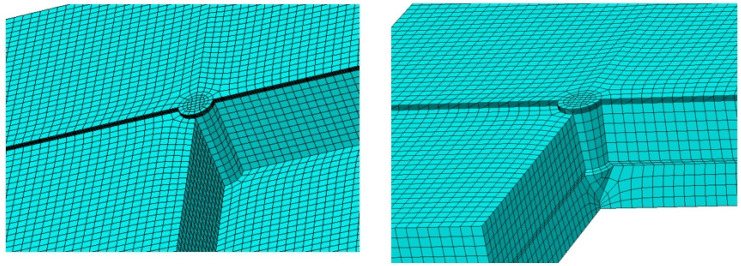
Mesh of models with one straight pin and one shaped pin.

A mesh refinement strategy was implemented around the pin area, as depicted in Figure 5. Appropriate boundary conditions and displacement loading were established to accurately simulate the quasistatic conditions of the lap shear tests. To ascertain the suitability of the mesh for simulation purposes, a convergence test was performed. In total, 170,960 hexahedral elements (identified by their element code in Abaqus) were utilized in the analysis.

The results obtained from the simulations corroborated the experimental data, ensuring the validity of the findings. The Von Mises and shear stress distributions were analyzed to inform the optimization of the dimensions and profile of the pin, which ultimately aims to reduce the stress values.

### 2.2. Experimental Study

The test samples were fabricated using a two-step process involving selective laser melting (SLM) technology. Initially, a pin was printed using a SLM machine (manufactured by Tianjin LiM Laser Technology Co., Ltd., Tianjin City, China) following the optimized shape derived from numerical simulation. Thereafter, the pin was inserted into a pre-drilled hole within a composite plate, with an adhesive layer placed between the metal and the composite to finalize the specimen assembly. Subsequently, these specimens underwent shear testing through displacement loading using a universal tensile testing machine, operating at a speed of 1 mm/min. Further details regarding the additive manufacturing process, the materials utilized, and the resultant test findings are elaborated in the subsequent sections.

#### 2.2.1. LiM-X260 Equipment

Selective Laser Melting (SLM) is recognized as a direct manufacturing technique for metal components, enabling the fabrication of parts with precise geometric dimensions and configurations directly from CADAutoCAD (Autodesk Computer Aided Design, version 2021) data. This process involves the additive layering of material.

In this study, the LiM-X260 (manufactured by Tianjin LiM Laser Technology Co., Ltd., Tianjin City, China) was employed as the SLM apparatus. The LiM-X260 is an industrial-grade 3D-printing device that utilizes metal powder, characterized by particle sizes ranging from 15 to 53 µm. During the forming process, the metal powder is thoroughly melted, facilitating metallurgical bonding. The key parameters of the printing process are summarized in Table 3.

#### 2.2.2. Three-Dimensional Printing Materials

**Figure 6:**
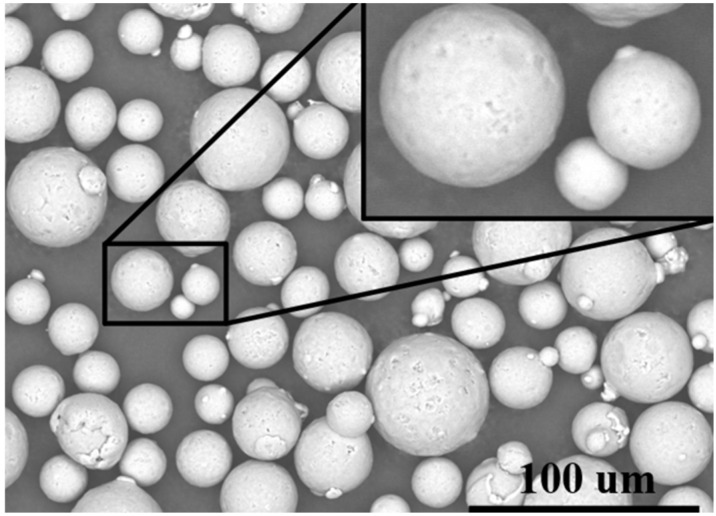
SEM morphology of AlSi10Mg powder.

**Figure 7:**
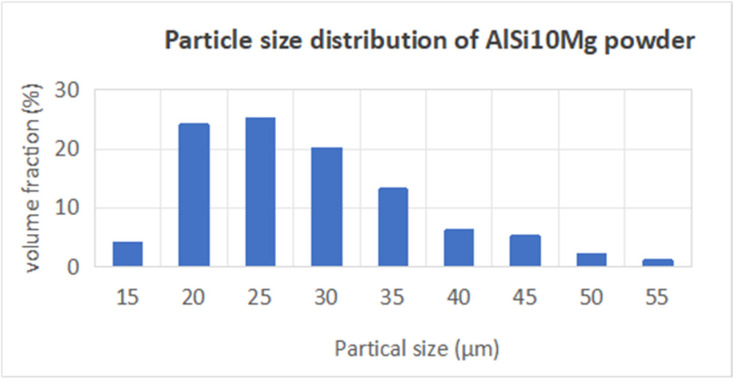
Particle size distribution of AlSi10Mg powder.

For this research, AlSi10Mg powder was selected as the material choice, due to its widespread application in aerospace engineering. The purchased powders (Nantong Jinyuan Intelligent Manufacturing Co., LTD, Nantong, China) had a particle size range from 15 µm to 53 µm, and their chemical compositions are shown in Table 4. Figure 6 clearly illustrates that the powder particles predominantly exhibit a spherical morphology. In Figure 7, the particle size distribution of the powder is presented, revealing that particles sized between 20 µm and 30 µm constitute a substantial fraction, accounting for nearly 70% of the total distribution.

#### 2.2.3. Carbon Fiber Plate and Adhesive

The carbon fiber plate (Jilin Chemical Fiber Group Co., Jilin, China) was constructed from 28 layers of woven prepreg plies, arranged in a cross-laying configuration at angles of 0° and 90°. The overall thickness of this plate measured 5 mm, and its mechanical properties are detailed in Table 1. To facilitate the joining process, a hole with a diameter of 1.9 mm and a depth of 3.43 mm was precisely drilled into the composite material.

For the adhesive bonding, an aviation-grade, two-component flexible AB adhesive (3M company, St. Paul, MN, USA) was employed. Following the manufacturer’s specifications, the mixing ratio of component B to component A was maintained at 2:3. Prior to the assembly, a thin layer of adhesive was uniformly applied to the contact surfaces of both the metal and composite plates. The metal plate was positioned so that its pin aligned precisely with the drilled hole in the composite. Subsequently, the assembly was subjected to deadweight pressing for a duration of 12 h at an ambient temperature, to ensure the proper curing of the adhesive.

## 3. Results and Discussion

### 3.1. Numerical Simulation Results

In the context of the Abaqus simulations, the coordinate system is defined such that the X-axis corresponds to direction 1, the Y-axis represents direction 2, and the Z-axis is identified as direction 3, as depicted in Figure 4b. Thus, S11 represents the normal stress in the X-axis, S22 denotes the normal stress in the Y-axis, and S12 indicates the shear stress in the Y direction in the plane.

**Figure 8:**
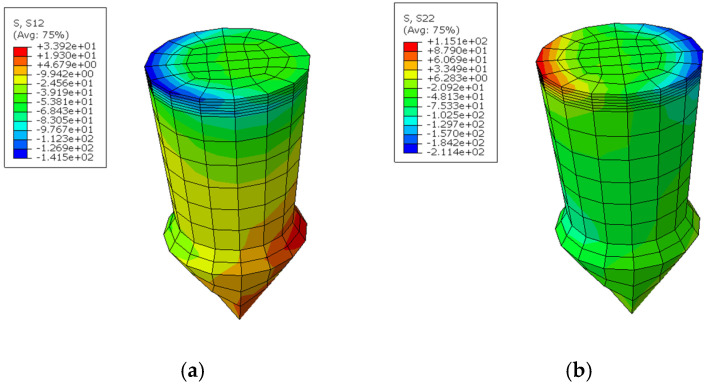
(**a**) Shear stress *s*_12_; (**b**) normal stress *s*_22_ in the pin.

In this study, the boundary conditions dictate that the left-hand end surface of the top plate is fixed, prohibiting any rotational movement. Conversely, the right-hand end surface of the bottom plate is subjected to a uniform distributed load oriented in the X-direction, while also maintaining a fixed rotational constraint. The analysis revealed that the peak shear and normal stresses within the pin occur at the cross-section situated between the base of the pin and the metal plate (Figure 8). The bearing stress, which represents the contact stress between the pin and the composite plate, is observed to increase in response to tensile loading. To further investigate the impact of pin geometry, a parametric study was executed, employing the range of values detailed in Table 5.

The results from this study facilitated a sensitivity analysis focused on the optimized pin geometry, particularly in relation to variations in the maximum and minimum stress values experienced by the pin. Sensitivity is quantified as the percentage difference between the maximum and minimum stresses relative to the maximum stress, as formulated in Equation (1). This sensitivity is notably affected by the geometric parameters of the pin. The results are listed in Table 6 for all dimensions except the tip angle, and as it displays, all geometric parameters (Figure 3) pose a clear influence on the compressive stress in the pin, particularly from diameter D1. However, the influence on the shear and tensile stresses is relatively moderate, except for the total height H of the pin, which has a clear effect on the shear stress. As observed in Figure 3, the influence of the tip angle is included in that of the total height, H.

(1) $$Sensitivity=\frac{\sigma_{max}-\sigma_{min}}{\sigma_{max}}\times 100\%$$

**Figure 9:**
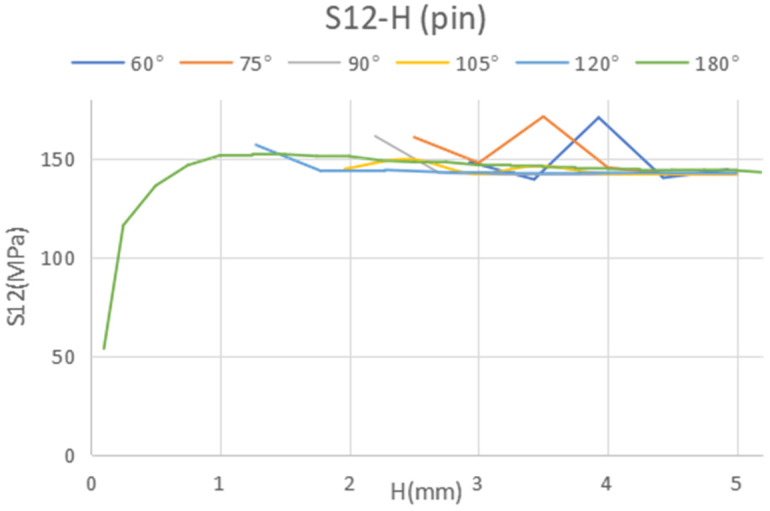
Shear stress in the pin bottom cross-section in terms of the pin height with different tip angle.

Considering the predominant failure mechanism for the pin, which is anticipated to be shear at the lower cross-section, we can determine the appropriate dimensions to minimize shear stress (Figure 9).

The optimized specifications for the geometry of the pin are detailed in Table 7.

### 3.2. Experimental Results

#### 3.2.1. Microscope Photo of the Pin

**Figure 10:**
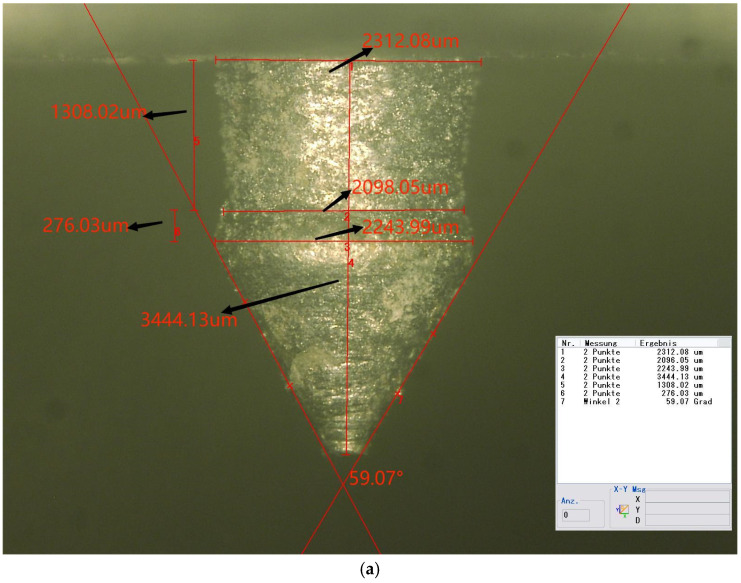

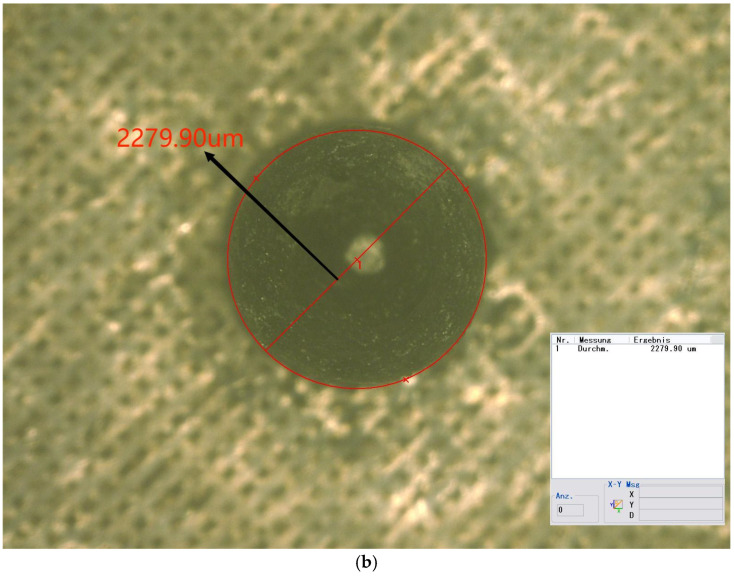
(**a**) Side view of the pin (50×); (**b**) top view of the pin (50×).

Three specimens were fabricated in the form of metal plates, each measuring 75 mm by 25 mm, with an attached pin. The pins were produced to closely resemble the optimized geometry. Figure 10 illustrates a microscope image of the pin. The measured dimensions and their deviations from the numerically optimized geometry are detailed in Table 8.

As indicated in the table, the neck height, H2, shows the largest deviation from the optimized values, at nearly 28%. Both D2 and H1 exhibit deviations of approximately 20%. The total height, H, and the tip angle are the most accurate dimensions, with errors of less than 1% for both. The remaining dimensions exhibit errors lightly exceeding 10%.

#### 3.2.2. Tests of Pinned Joint

Given its configuration as a hybrid joint incorporating an adhesive layer, it is essential to evaluate the joint’s strength performance. Initial assessments involved conducting lap shear tests to determine the strength of the adhesive in conjunction with the pin.

Before conducting the lap shear test, adhesive was applied to both the carbon fiber composite plate and the metal plate with a pin, over an area measuring 25 mm × 25 mm. Notably, the composite plate had a pre-drilled hole, which was positioned to precisely align with the pin on the metal plate. The two plates were then meticulously pressed together to ensure that the pin was inserted into the hole in the composite plate. According to the adhesive’s usage instructions, the two surfaces were allowed to set for 2 to 3 min before being pressed together and held under weight for 2 h.

After setting, the lap joint sample was tested using a universal tensile testing machine (CMT4304-Microcomputer Control Electronic Universal Testing Machine, MTS Systems, (China) Co., LTD, Shanghai, China). Displacement loading was applied, with a loading speed of 1 mm/min, until the metal and carbon fiber plates were separated, primarily in shear mode. However, the large deformation also resulted in some bending deformation during the test.

**Figure 11:**
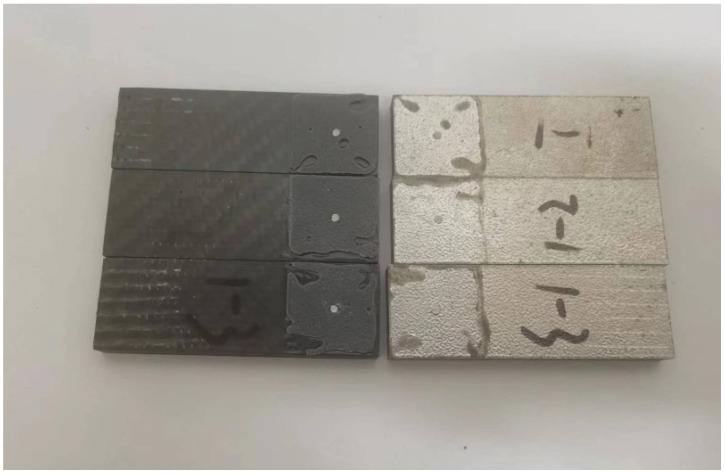
Tested sample and the single pin sheared at the base from the metal plate.

As illustrated in Figure 11, the post-test split plates reveal that the pin was sheared at the root to the metal plate, with its body embedded in the hole of the composite plate.

**Figure 12:**
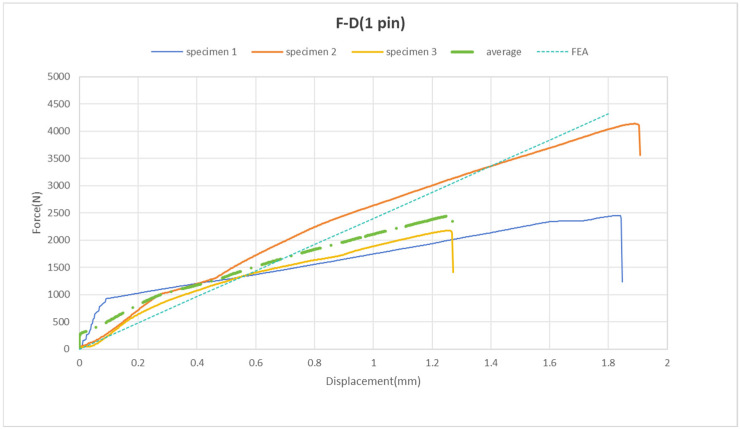
Force vs. displacement of pinned joint samples.

The pinned joints display a nearly linear increase in the force–displacement curve, as illustrated in Figure 12. It is observed that the total displacement reaches approximately 2 mm. The maximum force values at the failure points range from 2.2 kN to 4.2 kN, with an average of 2.5 kN (Figure 12).

The FEA conducted using simulation software shows that the force and displacement curves closely align with the experimental results (refer to Figure 12). Consequently, we can conclude that in the model with one pin, the experimental data agree with the simulated data. Thus, at this stage of the study, no attempts have been made to simulate failure within the FEA framework.

## 4. Conclusions

This research focuses on a numerical analysis of the joint’s strength and the optimization of a single pin. The joint between metal and composite materials is examined via finite element analysis, incorporating single-pin and adhesive-bonding layer joint models. The principal conclusions of this research are as follows:

(1) The overall height of the pin significantly influences the shear stress (S12) within the pin, with S12 decreasing as the height increases. However, this height has minimal impact on the axial stress (S22) in both the pin and the adhesive. For axial stress (S22), the shape of the pin, particularly its angle, is more influential. The optimal configuration for a single pin, involving a 60° angle and a total height of 3.43 mm, achieves the minimum shear stress;
(2) A microscopic photograph of a single pin produced via 3D-printing illustrates that the manufacturing accuracy is satisfactory;
(3) In the model featuring one pin, the experimental results align with the simulated data.

## 5. Future Work

The next phase of the study will focus on the design and optimization of joints involving multiple pins arranged in a matrix configuration. Both experimental tests and numerical simulations will be conducted.

In addition to optimizing the load bearing capacity of the hybrid joint, the study will address the failure mechanism to elucidate the ultimate strength of hybrid joints under various failure modes. This research aims to provide guidance on optimizing joining configurations between composites and metals for enhanced strength in the airline industry.

## Figures and Tables

**Table 1:** Material properties.

Section			Young’s Modulus, E (MPa)			Poisson’s Ratio			
Metal plate, pin (AlSi10Mg)			70,000			0.28			
Adhesive (EC-2216 B/A Gray)			2800			0.3			
Section	E_xx_ (MPa)	E_yy_ (MPa)	E_zz_ (MPa)	G_xy_ (MPa)	G_xz_ (MPa)	G_yz_ (MPa)	v_xy_	v_xz_	v_yz_
Carbon fiber plate	157,000	8500	8500	4200	4200	4200	0.35	0.35	0.35

**Table 2:** Geometry of the model.

Parameters	Value (mm)
Pin base diameter	2.00
Pin height	3.00
Metal plate thickness	5.00
Composite plate thickness	5.00
Adhesive thickness	0.25
Plate length	75.00
Overlap length	25.00

**Table 3:** Geometry of equipment.

Parameters	Value
Power of IPG, (W)	500
Laser spot diameter, (mm)	0.07–0.12
Scanning speed, (m/s)	≥7
Maximum molding size, (mm)	260 × 260 × 400

**Table 4:** Chemical composition of AlSi10Mg powder used in this work (in wt.%).

Characteristic	Value						Unit
Rest angle	29.32						°
Loose density	1.43						g/cm^3^
Chemical composition	Al	Si	Mg	Fe	O	Others	%wt
	89.24	10.01	0.331	0.119	0.056	<0.244	

**Table 5:** Range of values of the pin geometry used in the parametric study for the optimized pin shape.

Parameters	Range (°, mm)
α	60–180
D1	2–3
D2	1–2
H1	1–3
H2	0.1–0.3
H	≤5

**Table 6:** Sensitivity study on the pin’s geometric parameters.

Sensitivity Study (%)					
Section	D1	D2	H1	H2	H
*s*_12, Shear_	4.33	4.86	5.70	4.75	11.93
*s*_22, Tensile_	2.13	2.90	2.46	2.20	3.07
*s*_22, Compressive_	22.93	9.89	18.04	13.54	18.05

**Table 7:** Optimization of one pin (minimum stress of s_12_ and s_22_).

Section	Angle (°)	D1 (mm)	D2 (mm)	H1 (mm)	H2 (mm)	H (mm)
Best pin	60	2.00	1.60	1.50	0.20	3.43

**Table 8:** Measurements of pins and variations from the optimized geometry.

Specimens	D1	D2	D3 = D1	H	H1	H2	α	D-Top View
1	2310.77	1977.35	2250.67	3469.74	1250.76	297.35	60.87	2284.16
2	2285.48	2085.35	2250.73	3437.41	1313.39	264.03	59.99	2267.52
3	2268.04	1984.05	2296.06	3452.18	1216.05	268.02	60.37	2269.33
Average	2310.77	1977.35	2250.67	3469.74	1250.76	297.35	60.87	2284.16
Optimization	2000	1600	2000	3430	1500	200	60	2000
Error	12.59%	20.62%	11.73%	0.67%	−19.04%	27.66%	0.68%	12.04%

## Data Availability

The original contributions presented in this study are included in the article. Further inquiries can be directed to the corresponding authors.
